# Social Capital and Lifestyle Impacts on Mental Health in University Students in Colombia: An Observational Study

**DOI:** 10.3389/fpubh.2022.840292

**Published:** 2022-05-12

**Authors:** Lina Sotaquirá, Insa Backhaus, Paula Sotaquirá, Mónica Pinilla-Roncancio, Catalina González-Uribe, Raquel Bernal, Juan José Galeano, Natalia Mejia, Giuseppe La Torre, Elena M. Trujillo-Maza, Daniel E. Suárez, John Duperly, Andrea Ramirez Varela

**Affiliations:** ^1^School of Medicine, Universidad de los Andes, Bogotá, Colombia; ^2^Centre for Health and Society, Institute of Medical Sociology, Medical Faculty, University of Düsseldorf, Düsseldorf, Germany; ^3^Department of Public Health and Infectious Diseases, Sapienza University of Rome, Rome, Italy; ^4^School of Economics, Universidad de los Andes, Bogotá, Colombia; ^5^School of Economics, Universidad del Rosario, Bogotá, Colombia

**Keywords:** social capital, mental health, depressive symptoms, lifestyles, university students

## Abstract

**Introduction:**

For young adults, the first year of higher education represents a transition period into adulthood associated with an increased risk of developing depression, anxiety, and stress, contributing to deteriorating physical and mental health. The present study aimed to analyze the relationship between depressive symptoms and social capital and lifestyles among Colombian university students.

**Methods:**

In 2020, a longitudinal repeated measures study was conducted on first year students at Universidad de los Andes in Bogota, Colombia. The study was conceptualized and approved by the university before the COVID-19 pandemic appeared. Each student completed a self-administered questionnaire including questions on sociodemographic characteristics, depressive symptoms, perceived stress, social capital, and lifestyles. The study's pilot was conducted in November 2019, and the two measurement points were in January 2020 (wave 1, before the COVID-19 pandemic was declared) and in August 2020 (wave 2, during the COVID-19 pandemic). A binary logistic regression analysis was performed to assess the relationship between depressive symptoms, perceived stress, social capital, and lifestyles.

**Findings:**

A total of 609 first year students (response rate = 58.11%) participated in wave 1, and 42% of the participants showed signs of clinically relevant depressive symptoms. In wave 2, despite the difficulties encountered in collecting data due to the COVID-19 pandemic, 216 students from wave 1 participated (35.47%). An increase in a sedentary lifestyle was observed (31.49%). We found that cognitive and behavioral social capital levels decreased by 12.03 and 24.54%, respectively. In addition, we observed a 6.5% increase in students with clinically relevant depressive symptoms compared to wave 1. A low level of behavioral [*OR*: 1.88; 95% CI (1.16, 3.04)] social capital was associated with clinically relevant depressive symptoms.

**Conclusion:**

The health of university students continues to be a public health concern. The study suggests that social capital may play an important role in preventing depressive symptoms. Therefore, universities should put effort into programs that bring students together and promote the creation of social capital.

## Introduction

It is widely recognized that first year university students are exposed to various stressors when leaving the parental home and starting a new independent life (1). Generally, beginning college is associated with increased responsibilities and is coupled with a period of rising insecurities and emotional pressure (2, 3). Many students also experience a change in their overall lifestyles and social activities, including a decrease in physical activity, frequent use of alcohol, changes in diet, and increased stress levels (2). Furthermore, students often face a competitive environment and the need to create new social networks (4). These factors shape the student population and increase their susceptibility to mental health problems (2, 5).

With the onset of the COVID-19 pandemic, a potential additional stressor arose for students. In Colombia, for instance, a state of emergency was declared on March 17, 2020, after the first COVID-19 cases were detected on March 6, 2020, which resulted in university life changing immediately. Students were asked to stay away from campus, study remotely, and attend virtual classes. The measures taken to contain the spread of the virus have impacted everyone worldwide, but one of the most affected groups was probably the student population (6, 7). The closures of educational institutions and the shift to online education have caused changes in students' social life and lifestyles due to social isolation and fewer activities with peers (8, 9). Recent data show that slightly more than half of the college students experienced worsening mental health in 2020, including increased stress, anxiety, and depression (10).

Several factors have been proposed to reduce the effect of crises on health. One crucial factor discussed is social capital (11). For instance, Pitas and Ehmer (11) demonstrate that communities with high social capital respond more effectively than those with low levels of social capital. Social capital, however, has not only been shown to have beneficial effects on health during crises, but also in general. The above is consistent with what is proposed in the social determinants of health model, which explains how social, economic, and environmental factors determine the health status of individuals or populations (12, 13).

Regarding social capital, there is a growing literature showing that higher levels of social capital can benefit health (14–16). Studies that have analyzed the association between social capital and lifestyles, for instance, have shown that strengthening social networks can eradicate risky behaviors in the general population (14). In addition, higher levels of cognitive social capital have been associated with a lower risk of developing major depression (15) and with higher levels of regular, moderate, or vigorous physical activity (14–16).

Studies assessing the association between social capital and young adults' health, especially first year university students, are currently limited globally and in Colombia (17). Furthermore, most studies on mental health and social capital have been done in school, and adult populations in developed countries, and only a very few studies have evaluated this association in developing countries, such as Colombia (18–22). Additionally, most studies used cross-sectional data, which does not allow to make inferences about causality. Therefore, the present study aimed to explore the relationship between depressive symptoms and social capital and lifestyles through a repeated measure study, including two measurements 6 months apart during the first year of university studies. The COVID-19 pandemic started in the middle of our first wave of data collection.

## Research Methods

### Study Design

A longitudinal repeated measures study was performed to analyze the relationship between depressive symptoms, social capital, and lifestyles (stress, physical activity, alcohol consumption, smoking, diet, and sleep) in first year students at Universidad de los Andes Bogotá, Colombia. This study was conceptualized and approved by the university before the COVID-19 pandemic appeared. This is one of the sites of the SPLASH “Social Capital And Students' Health—An international two-wave panel study” multicentric multinational study, that involves since 2018 Germany, Australia, Brazil, China, South Korea, United States, Malaysia, Switzerland, Italy, Oman, and Taiwan (17).

### Study Setting

Data collection was conducted in South America in Bogota, Colombia's capital city. The pilot study was in November 2019. Data were collected at the beginning and the end of the first semester. An academic semester in a Colombian university lasts 6 months; therefore, this is the reason for selecting the start and end of this period to conduct the study assessments. The first wave of data collection was carried out in person from January 21 to February 24, 2020. A self-administered questionnaire was provided to first-year students with the accompaniment of the research team in their classrooms.

Data collection for wave two was scheduled for August 2020, and the same methodology used for wave one was to be employed. However, due to the COVID-19 pandemic, it was necessary to adjust this methodology because all classes were online, and the country was in lockdown. Therefore, an e-mail including a survey link was sent to all participants with weekly reminder e-mails. The second study wave was conducted from August 12 to November 27, 2020.

### Sample Size

The study aimed to evaluate the relationship between depressive symptoms and social capital. The sample size was calculated based on the prevalence of depressive symptoms in Colombia (26%) (23). Thus, based on this data, and with a sensitivity of 95%, a margin of error of no more than ±5%, the minimum sample size required for this study was 290 students, adjusted for 20% of incomplete responses, and 30% loss to follow-up rate, the final minimum required sample size was 580 students.

### Participants

The target population consisted of 1,048 first year students from Universidad de los Andes. For participant selection, an e-mail was sent to each of the 43 academic program coordinators with detailed information about the project, requesting permission to talk to students during class and inviting them to participate in the study. Twenty-five coordinators responded and allotted 30 min in the introductory courses to administer the questionnaire. Participants were aged between 15 and 24 years, were in their first semester, had begun their studies at the Universidad de los Andes in the 2020–2021 academic period, and were enrolled full-time. Participants who had re-entered the university were excluded from the study. In the first contact with the students, the study procedures were explained in detail and the importance of their participation in both surveys one at the beginning and another at the end of the semester was mentioned.

### Variables and Measurements

Students were invited to complete a 58-question self-administered questionnaire lasting 25–30 min. It assessed four components: sociodemographic characteristics, social capital, mental health, and lifestyles. Previously validated questionnaires were obtained from a rigorous review of the literature, which has also been used in the annual international survey or SPLASH that examines mental health and related factors among university students worldwide. All were translated into Spanish to be applied to students. Where applicable, we used the same questions as applied in the study by Backhaus et al. (17), allowing for comparisons between our study and the study of Backhaus et al. (17). A detailed description of each of the variables is presented below.

#### Dependent Variable

##### Depressive Symptoms

The simplified Beck Depression Inventory (BDI-S) was used (24, 25). The BDI-S or BDI-V measures the frequency of depressive symptoms (e.g., hopelessness, irritability) as well as physical symptoms (e.g., fatigue, loss of appetite). The modified inventory has 20 questions and measures the severity of depressive symptoms through a 6-point Likert scale ranging from 0 (never) to 6 (almost always). The inventory asks how current life feels with statements such as “I am sad” or “I look discouraged toward the future.” The score ranges from 0 to 100 (25). According to the established cut-off points, a BDI-S score ≥ 35 suggests the presence of clinically relevant depressive symptoms (24, 25). The assessment of suicidal thinking was based on item 9 of the BDI-S, which asks if the person has had suicidal thoughts and has been considered a robust predictor of suicide attempts (26).

#### Independent Variables

##### Sociodemographic Characteristics

This component had 13 questions, inquiring about the participant's age, nationality, marital status, the program in which he/she was enrolled, data on the participant's parents. Regarding socioeconomic status, the socioeconomic stratum of the participant's place of residence and perception of sufficient income to cover monthly expenses were asked. These components were evaluated to determine their effect on the other study variables (27).

##### Social Capital

We assessed two dimensions of cognitive and behavioral social capital using the Integrated Questionnaire for Measuring Social Capital by the World Bank (28). Cognitive social capital was composed of five questions, evaluating the components of trust, solidarity, lending, and cooperation. The questions were measured on a 5-point Likert scale (0 = strongly agree and 5: strongly disagree) and one with binary option (0 = you cannot be too careful and 1 = people can be trusting). Behavioral social capital was composed of nine questions of binary choice (yes or no) or Likert scale, assessing the components of Groups and networks, collective action, and cooperation. The obtained scores were divided into low and high levels of social capital. The behavioral score ranged from 0 to 10, with low levels being a score of <5. The cognitive score ranged from 0 to 22, with low levels being <12.

##### Self-Rated Health

Students were asked how they perceived their health. Answers were divided into fair/poor health vs. good/very good/excellent.

##### Perceived Stress

Perceived stress was measured using the Cohen Perceived Stress Scale, a 10-item scale that assesses the extent to which a respondent finds life situations stressful. The questions focus on how respondents find their lives unpredictable, uncontrollable, and overloaded. It uses a 5-point Likert response format ranging from 0 (*never*) to 4 (*very often*). Scores ranged from 0 to 40, with lower stress levels <14, moderate levels from 14 to 26, and high levels > 27 (29).

##### Lifestyles

###### Alcohol Consumption.

Alcohol consumption was measured using the Audit-C questionnaire (30). The Audit-C consists of three questions, which assess the frequency with which participants consume alcoholic beverages. Score can range from 0 to 12 with higher scores indicating hazardous drinking (30).

##### Smoking

Smoking status was assessed by using six questions developed by the WHO. These questions asked whether the participant smoked, and if so, how often, in what way, and whether he or she had reduced or stopped smoking. For the analysis students were divided into non-smokers and smokers (31).

##### Physical Activity

The validated IPAQ instrument was used, which consisted of seven questions that evaluated the frequency with which the participants performed physical activity of low, moderate, and vigorous intensity. For the analysis, we calculated the METs score, and based on their score, determined whether a student performed low, moderate, or vigorous physical activity. Students with a score of <600 MET were classified as performing low-intensity physical activity, between 600 and 2,999 MET, moderate physical activity, and ≥3,000 MET vigorous physical activity (32).

##### Eating and Sleeping

This questionnaire included two questions that evaluated if the participant's diet was balanced and if the participant slept well. It uses a 5-point Likert-type response format ranging from 0 (almost never) to 4 (almost always).

### Data Analysis Strategy

Descriptive analyses were conducted. The main outcome was dichotomized according to BDI-S scores: not clinically depressed (<35) vs. clinically relevant depression (≥35). Statistical analysis was carried out using Stata 16 software. A descriptive analysis of each variable was performed to characterize the sample and determine the levels of social capital, depressive symptoms, perceived stress, and lifestyles in the sample evaluated. The variables associated with depressive symptoms were analyzed using Pearson's chi-square test, presenting relative and absolute frequencies. The null hypothesis was that the independent variables did not have a statistically significant association with the dependent variable of depressive symptoms, which was rejected if the *p*-value was ≤ 0.05.

**Figure 1:**
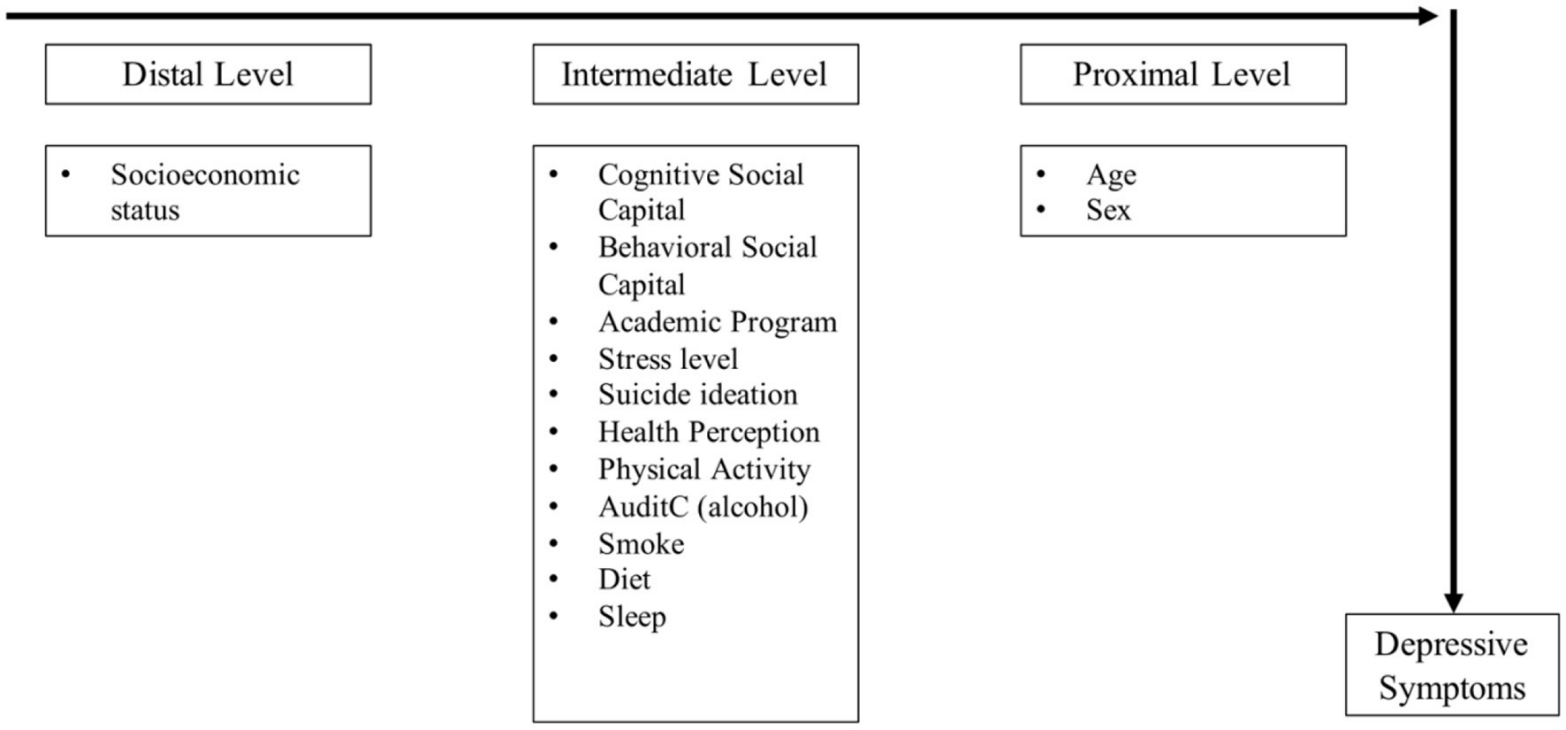
The hierarchical conceptual model of factors associated with depressive symptoms.

A binary logistic regression was carried out to estimate the association between clinically relevant depressive symptoms, social capital, perceived stress, and other lifestyles variables. The relationship between clinically relevant depressive symptoms and independent variables: Demographic data, levels of social capital, stress, and lifestyles (physical activity, alcohol, and tobacco consumption, diet, and sleep) were estimated using a hierarchical conceptual model with backward elimination of the variables. At each level of analysis, variables with a *p*-value ≤ of 0.20 were retained in the model. The final model included variables that were statistically associated with the outcome (see Figure 1). The level of significance was set at 0.05, and a confidence level of 95% was chosen.

The data set collected in wave 1, and 2 was analyzed as a panel data, which consists of repeated observations over time, in this case, observations of each of the participants at two points in time, wave 1 and wave 2. To assess changes in outcomes between waves 1 and 2, we applied McNemar's test for categorical variables paired with two categories and the adjusted McNemar's test, Symmetry (asymptotic), for categorical variables paired with more than two categories. This was intended to test the hypothesis that the changes observed between waves 1 and 2 were not due to chance. The level of significance was set at 0.05. Changes in mental health, lifestyles, and social capital were obtained by analyzing the panel data from the participants' first and second waves. A least-squares model with fixed effects was chosen to control for any individual-specific attributes that do not vary across time. Coefficients with 95% confidence intervals were obtained. The significance level was set at 0.05.

## Findings

### Sample Characteristic of Participants: Wave 1 and Wave 2

**Figure 2:**
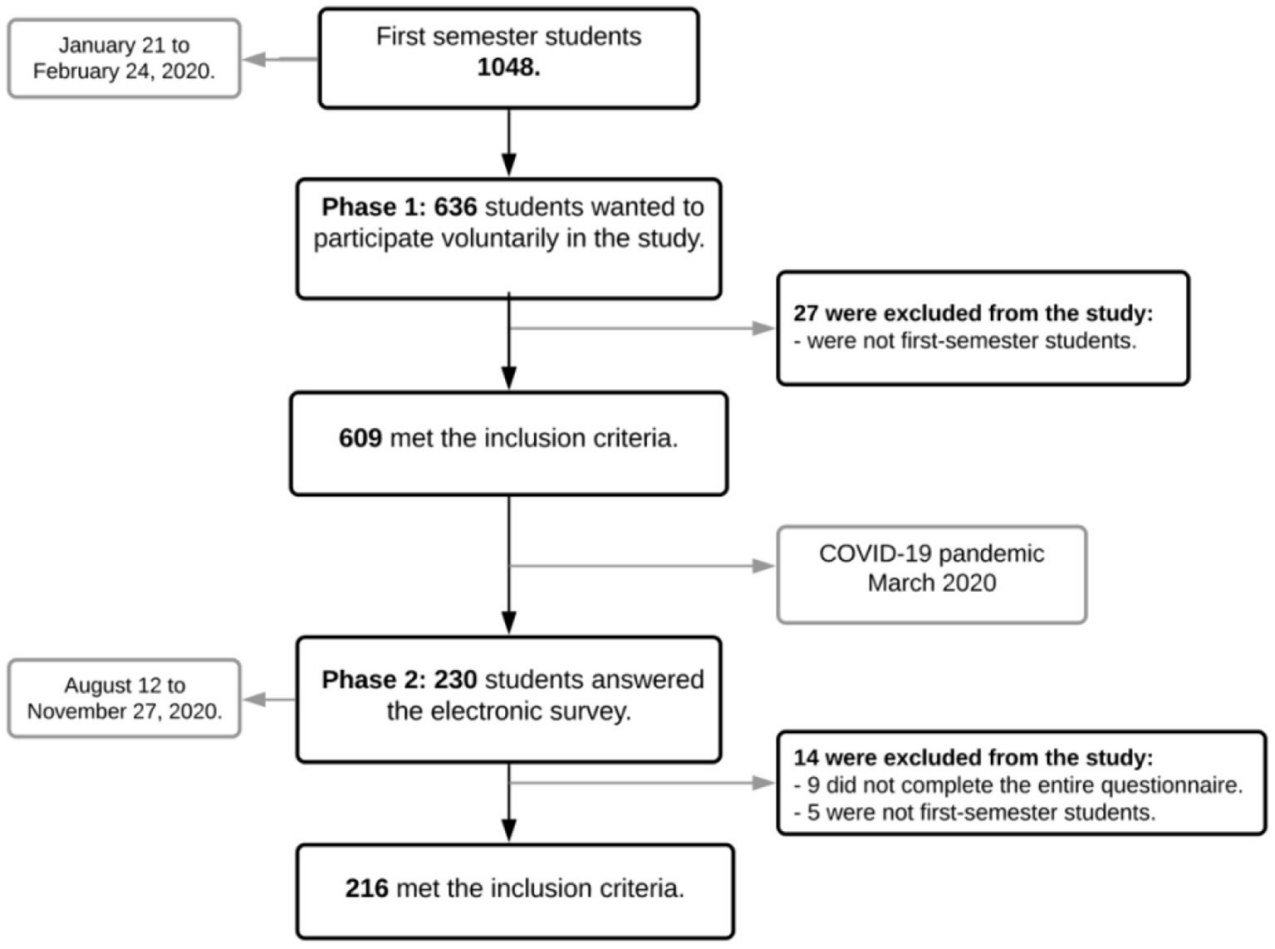
Collection wave flowchart.

In the study's first wave, 630 students participated; 21 students had to be excluded as they did not meet the inclusion criteria (i.e., non-first year students). One student did not answer questions related to behavioral social capital and was subsequently removed from the analysis. In wave 2, 230 students participated; 14 students had to be excluded (9 did not complete the entire questionnaire, and five did not meet the inclusion criteria). Unlike wave 1, the questionnaire could not be applied in person but had to be sent by mail due to the pandemic. Thus, 609 data from wave 1 (response rate = 58.11%) and 216 from wave 2 (representing 35.47% of respondents from wave 1) were used for the statistical analysis (Figure 2). Due to the loss of follow-up in wave 2, the results of longitudinal changes have to be interpreted with caution.

The mean age of the participants in wave 1 was 17.25 (SD: ±1), and more men (63.22%) than women participated. Most of the students were enrolled in an engineering program. The percentage of students living at home with their parents was 54.19%. A BDI-S above 35, indicating the presence of clinically relevant depressive symptoms, was observed in 42.20% of participants in wave 1, and 24.14% of these students indicated that they have thought about suicide at least once (Table 1).

**Table 1:** Sociodemographic characteristics and lifestyles of university students with and without depressive symptoms.

	**Total sample**		**Not clinically relevant (BDI-S** **<** **35)**		**Clinically relevant (BDI-S** **>** **35)**		
	***n***	**%**	***n***	**%**	***n***	**%**	***p*-value**
Total	609	100	352	57.80	257	42.20	
Sex							<0.001*
Male	385	63.22	250	64.94	135	35.06	
Female	224	36.78	102	45.54	122	54.46	
Age							0.276
15–17	407	66.83	229	56.27	178	43.73	
18–24	202	33.17	123	60.89	79	39.11	
Socioeconomic status							0.847
Low	265	43.51	152	57.36	113	42.64	
High	344	56.49	200	58.14	144	41.86	
Housing first wave							0.025
Parents' home	330	54.19	205	62.12	125	37.88	
Relative's house	72	11.82	30	41.67	42	58.33	
University residence	95	15	51	53.68	44	46.32	
Rented house	90	14.78	52	57.78	38	42.22	
Other	22	3.61	14	63.64	8	36.36	
Program							<0.001*
Engineering	351	57.64	231	65.81	120	34.19	
Social sciences	150	24.63	69	46.00	81	54.00	
Sciences	38	6.24	16	42.11	22	57.89	
Health sciences	37	6.08	22	59.46	15	40.54	
Directed studies	30	4.93	12	40.00	18	60.00	
Architecture and arts	3	0.49	2	66.67	1	33.33	
IPAQ physical activity categories							0.001*
Low physical activity level	335	55.01	171	51.04	164	48.96	
Moderate physical activity level	231	37.93	151	65.37	80	34.63	
Vigorous physical activity level	43	7.06	30	69.77	13	30.23	
Sedentariness: time spent sitting in a working day							0.003*
1–4 h	184	30.21	121	65.76	63	34.24	
5–9 h	327	53.69	187	57.19	140	42.81	
10 h or more	98	16.09	44	44.90	54	55.10	
Heavy episodic drinking							0.533
Low-risk drinkers	430	70.61	252	58.60	178	41.40	
High-risk drinkers	179	29.39	100	55.87	79	44.13	
Smoking status							0.225
Non-smoker	595	86.04	308	58.78	216	41.22	
Ever smoker	85	13.96	44	51.76	41	48.24	
Balanced diet							<0.001*
Yes	369	60.59	243	65.85	126	34.15	
No	240	39.41	109	45.42	131	54.58	
Sleep: do you sleep well and feel rested?							<0.001*
Yes	212	34.81	158	74.53	54	25.57	
No	397	65.19	194	48.87	203	51.13	
Self-rated health							<0.001*
Good	574	94.25	347	60.45	227	39.55	
Fair/poor	35	5.75	5	14.29	30	85.71	
Suicide ideation							<0.001*
No suicidal ideation	462	75.86	325	70.35	137	29.65	
Yes, at least once	147	24.14	27	18.37	120	81.63	
Perceived stress							<0.001*
Low stress	7	1.15	6	85.71	1	14.29	
Moderate stress	562	92.28	337	59.96	225	40.04	
High stress	40	6.57	9	22.50	31	77.50	
Social capital: cognitive dimension							0.01*
Low cognitive social capital level	204	33.50	103	50.49	101	49.51	
High cognitive social capital level	405	66.50	249	61.48	156	38.52	
Social capital: behavioral dimension^a^							0.001*
Low behavioral social capital level	106	17.43	46	43.40	60	56.60	
High behavioral social capital level	502	82.57	305	60.76	197	39.24	
Bonding bridging							0.207
Bridging social capital	407	66.83	228	56.02	179	43.98	
Bonding social capital	202	33.17	124	61.39	78	38.61	

*N = 609.*

a *The total does not add up to 609 due to missing data.*

* *p ≤ 0.05*.

### Factors Associated With Depressive Symptoms

Tables 2, 3 show the results of the unadjusted and adjusted regression model. Some of the findings are described below. Being female [*OR*: 1.90, 95% CI (1.30, 2.75)] low levels of behavioral social capital [OR: 1.88, 95% CI (1.16, 3.04)] fair/poor health [*OR*: 6.68, 95% CI (2.46, 18.18)], and high levels of stress [*OR*: 4.04, 95% CI (1.78, 9.17)], were associated with clinically relevant depressive symptoms. Among lifestyle factors, not sleeping well [*OR*: 3.09, 95% CI (2.07, 4.60)] was a significant risk factor. Regarding physical activity, moderate [OR: 0.66, 95% CI (0.45, 0.97)] physical activity was a protective factor for the presence of depressive symptoms.

**Table 2:** Binomial logistic regression showing the crude risk of depressive symptoms among university students.

	**Clinically relevant depressive symptoms (BDI-S** **≥35)**		
	**Unadjusted *OR***	**95% CI**	***p*-value**
**Proximal level: sociodemographic characteristics**			
Sex			
Female	2.21	(1.58, 3.09)	<0.001*
Male	1.00		
Age			
15–17	1.21	(0.86, 1.70)	0.277
18–24	1.00		
Socioeconomic status			
Low	1.03	(0.75, 1.43)	0.847
High	1.00		
Social capital: cognitive dimension			
Low cognitive social capital level	1.57	(1.11, 2.20)	0.010*
High cognitive social capital level	1.00		
Social capital: behavioral dimension^a^			
Low behavioral social capital level	2.02	(1.32, 3.09)	0.001*
High behavioral social capital level	1.00		
**Intermediate level**			
Self-rated health			
Fair/poor	9.17	(3.51, 23.99)	<0.001*
Good	1.00		
Perceived stress			
Low stress	0.25	(0.30, 2.09)	0.200
Moderate stress	1.00		
High stress	5.16	(2.41, 11.04)	<0.001*
Smoking status			
Ever smoker	1.32	(0.84, 2.10)	0.226
Non-smoker	1.00		
Heavy episodic drinking			
High-risk drinkers	1.12	(0.79, 1.59)	0.533
Low-risk drinkers	1.00		
Sleep: do you sleep well and feel rested?			
No	3.06	(2.12, 4.42)	<0.001*
Yes	1.00		
IPAQ physical activity categories			
Low physical activity level	1.00		
Moderate physical activity level	0.55	(0.39, 0.78)	0.001*
Vigorous physical activity level	0.45	(0.23, 0.90)	0.023*

*N = 609.*

*OR, odds ratio.*

a *The total does not add up to 609 due to missing data.*

* *p ≤ 0.05*.

**Table 3:** Results for binomial logistic regression, displaying adjusted odds ratios and 95% confidence intervals for depressive symptoms.

	**Model**		
	**Adjusted *OR***	**95% CI**	***p*-value**
**Proximal level: sociodemographic characteristics**			
Social capital: cognitive dimension			
Low cognitive social capital level	1.25	(0.86, 1.83)	0.244
High cognitive social capital level	1.00		
Social capital: behavioral dimension ^a^			
Low behavioral social capital level	1.88	(1.16, 3.04)	0.010*
High behavioral social capital level	1.00		
Age			
15–17	1.12	(0.76, 1.65)	0.594
18–24	1.00		
Sex			
Male	1.00		
Female	1.90	(1.30, 2.75)	0.001*
**Intermediate level**			
Self-rated health			
Good	1.00		
Fair/poor	6.68	(2.46, 18.18)	<0.001*
Perceived stress			
Low stress	0.26	(0.28, 2.31)	0.225
Moderate stress	1.00		
High stress	4.04	(1.78, 9.17)	0.001*
Sleep: do you sleep well and feel rested?			
Yes	1.00		
No	3.09	(2.07, 4.60)	<0.001*
IPAQ physical activity categories			
Low physical activity level	1.00		
Moderate physical activity level	0.66	(0.45, 0.97)	0.032
Vigorous physical activity level	0.59	(0.28, 1.26)	0.175
Intraclass correlation coefficient	0.21	(0.13, 0.35)	<0.001*

*N = 609.*

*OR, odds ratio.*

a *The total does not add up to 609 due to missing data.*

* *p ≤ 0.05*.

### Longitudinal Changes

Due to COVID-19 pandemic lockdowns and mobility restrictions, the data collection methodology for wave 2 had to be changed from in person to virtual. As mentioned above, the response rate decreased, impeding us from estimating the associations with adequate statistical power. Therefore, the following results have to be interpreted with caution and in light of the valuable information that was collected during an unprecedented global public health crisis such as the COVID-19 pandemic.

Table 4 presents the Sociodemographic Characteristics and Health-Related Behaviors of the 216 students who participated in the two waves. The average age of the students was 17.76 (SD: ±1), 57.41% of the students were men, 52.31% had a high socioeconomic status, and 53.24% were from the engineering program. When comparing these students' responses with those of wave 1, the proportion of students with clinically relevant depressive symptoms increased by almost 7%. The proportion of students living at home with their parents increased by 29.16%.

**Table 4:** Sociodemographic characteristics and health-related behaviors of university students with and without depressive symptoms.

	**Wave 1**		**Wave 2**			
	***n***	**%**	***n***	**%**	**Difference**	***p*-value^**a**^**
**Total**	**216**	**100**	**216**	**100**		
Sex						
Male	124	57.41	124	57.41		
Female	92	42.59	92	42.59		
Age	17.19 + 0.85^b^	15–21^c^	17.76 + 0.92^b^	16–22^c^	0.57 + 0.80^b^	<0.001*
15–17	154	71.30	97	44.91		
18–24	62	28.70	119	55.09	26.39	<0.001*
Socioeconomic status						
Low	103	47.69	103	47.69		
High	113	52.31	113	52.31		
Housing						
Parents' home	117	54.17	180	83.33	29.16	<0.001*
Relative's house	24	11.11	21	9.72	−1.39	
University residence	35	16.20	2	0.93	−15.27	
Rented house	33	15.28	13	6.02	−9.26	
Other	7	3.24			−3.24	
Program						
Engineering	115	53.24	115	53.24		
Social sciences	60	27.78	60	27.78		
Sciences	20	9.26	20	9.26		
Health sciences	13	6.02	13	6.02		
Directed studies	6	2.78	6	2.78		
Architecture and arts	2	0.93	2	0.93		
IPAQ physical activity categories						
Low physical activity level	124	57.41	152	70.37	12.96	0.001*
Moderate physical activity level	84	38.89	60	27.78	−11.11	
Vigorous physical activity level	8	3.70	4	1.85	−1.85	
Sedentariness: time spent sitting in a working day						
1–4 h	50	23.15	11	5.09	−18.06	<0.001*
5–9 h	124	57.41	95	43.98	−13.43	
10 h or more	42	19.44	110	50.93	31.49	
Heavy episodic drinking						
Low-risk drinkers	164	75.93	182	84.26	8.33	0.004*
High-risk drinkers	52	24.07	34	15.74		
Smoking status						
Non-smoker	195	90.28	205	94.91	4.63	0.018*
Ever smoker	21	9.72	11	5.09		
Balanced diet						
Yes	132	61.11	150	69.44	8.33	0.016*
No	84	38.89	66	30.56		
Sleep: do you sleep well and feel rested?						
Yes	69	31.94	70	32.41	0.47	0.908
No	147	68.06	146	67.59		
Self-rated health						
Good	204	94.44	205	94.91	0.47	0.763
Fair/poor	12	5.56	11	5.09		
Depressive symptoms						
Not clinically relevant (BDI-S <35)	110	50.93	96	44.44		
Clinically relevant (BDI-S > 35)	106	49.07	120	55.56	6.49	0.061
Suicide ideation						
No suicidal ideation	156	72.22	155	71.76	0.46	0.869
Yes, at least once	60	27.78	61	28.24		
Perceived stress						
Low stress	2	0.93	2	0.93		0.891
Moderate stress	192	88.89	189	87.50	−1.39	
High stress	22	10.19	25	11.57	1.38	
Social capital: cognitive dimension						
Low cognitive social capital level	60	27.78	86	39.81	12.03	0.001*
High cognitive social capital level	156	72.22	130	60.19		
Social capital: behavioral dimension						
Low behavioral social capital level	42	19.44	95	43.98	24.54	<0.001*
High behavioral social capital level	174	80.56	121	56.02		
Bonding bridging						
Bridging social capital	147	68.06	186	86.11	18.05	<0.001*
Bonding social capital	69	31.94	30	13.89		

*N = 216.*

a *McNemar test for paired categorical variables with two categories and adjusted McNemar test, Symmetry (asymptotic) for paired categorical variables with more than two categories.*

b *Mean + SD.*

c *Range.*

* *p ≤ 0.05*.

Regarding lifestyles, there was a 13% increase in the number of students who reported doing low-intensity physical activity and a 31.49% increase in students who reported sitting for 10 h or more. Clinically relevant depressive symptoms increased by 6.49%, and stress levels increased by only 1.38%. Concerning the levels of social capital, cognitive capital decreased by 12.03%, and behavioral capital decreased by 24.54%.

Evaluating the results of the least-squares model with fixed effects, we found that only two of the assessed variables showed a statistically significant association with depressive symptoms. While sleeping well decreased the risk of suffering from depressive symptoms by 14 percentage points, smoking increased the risk of suffering from depressive symptoms by 23 percentage points (see Table 5).

**Table 5:** Results of the least-squares model with fixed effects, showing coefficients and 95% confidence intervals for depressive symptoms.

	**Model**		
	**Coefficient**	**95% CI**	***p*-value**
**Proximal level: sociodemographic characteristics**			
Social capital: cognitive dimension			
High cognitive social capital level	0.03	(−0.09, 0.16)	0.600
Social capital: behavioral dimension			
High behavioral social capital level	−0.08	(−0.20, 0.46)	0.220
Age			
18–24	0.05	(−0.10, 0.19)	0.530
**Intermediate level**			
Self-rated health			
Good	−0.01	(−0.31, 0.28)	0.933
Perceived stress			
Moderate stress	−0.13	(−0.62, 0.36)	0.608
High stress	0.06	(−0.45, 0.57)	0.814
Sleep: do you sleep well and feel rested?			
Yes	−0.15	(−0.26, −0.03)	0.012
IPAQ physical activity categories			
Moderate physical activity level	−0.11	(−0.26, 0.04)	0.144
Vigorous physical activity level	0.19	(−0.12, 0.50)	0.227
Smoking status			
Ever smoker	0.23	(−0.01, 0.47)	0.051
Intraclass correlation coefficient	0.71	(0.14, 1.28)	0.015*

*N = 216.*

* *p ≤ 0.05*.

## Discussion

This is the first longitudinal study assessing clinically relevant depressive symptoms and the relationship with levels of social capital and lifestyles among university students. Data collection had to be held during the COVID-19 pandemic. The main results included a clinically relevant depressive symptom rate of 42.2%, an 88% higher risk of depressive symptoms among participants with low levels of behavioral social capital, 90% higher risk in women, 300% higher risk in participants with high levels of perceived stress, and 200% higher risk in participants who do not sleep well. Regular moderate physical activity was a protective factor against depressive symptoms, sleeping well decreased the risk of suffering depressive symptoms by 14%, and smoking increased that risk by 23%.

When comparing results with the study of Backhaus et al. (17) and the prevalence rates of other countries, the rate of depressive symptoms in Colombia is not as low as in Belgium (22%) and not as high as Brazil's (86%) but similar to rate detected in Oman (41%) and Taiwan (40%).

Being a female student, having lower behavioral social capital, high levels of perceived stress, and suffering from poor sleep quality significantly increased the risk of depressive symptoms. The findings regarding social capital are in line with previous findings demonstrating the beneficial effect of social capital on mental health (17, 33–35). Individuals with high behavioral social capital levels may receive more outstanding social support from their social networks. Therefore, it is possible that experiencing less social isolation results in a lower risk of depression (35, 36). Based on some published studies, high levels of social capital have been a protective factor for mental health by preventing not only depression but also anxiety and by favoring healthy lifestyles such as regular physical activity, a balanced diet, and less substance use (21, 33, 37). The literature also highlights the protective role of social capital in reducing stress levels, a known risk factor for depression (38).

The finding regarding gender differences also corresponds with those reported in other studies which suggested that, due to sociocultural factors, women are more likely to express their emotions and, therefore, more readily admit when they have depressive feelings (36).

Another factor associated with clinically relevant depressive symptoms was perceived stress. We found that most of the sample (98.85%) reported having moderate to high stress levels. Of the students who reported high stress levels, 77.50% had a BDI-S > 35. Students often experience stress related to their academic careers, such as lack of time to complete academic activities, exposure to classwork, and academic overload (39). These academic stressors are a subset, among other factors that generate high stress levels in this population. First year students, in particular, are more likely to report higher stress rates due to lack of time for other activities and academic overload. Although upper-level university students continue to perceive these factors as stressors, they attenuate to some extent over time (38).

Regarding lifestyle results, we found that <10% of the students reported participating in vigorous exercise. Of the participants who only engaged in a low-intensity activity, such as walking, almost half (48.96%) reported having depressive symptoms, indicating that moderate and vigorous physical exercise can be a protective factor. These findings are consistent with those reported in the SPLASH study (17). When comparing the results of wave 1 and wave 2, we observed a decrease in physical activity among participants and a corresponding increase of 31.49% who reported sitting for more than 10 h a day. This shift from an active to a sedentary lifestyle could be explained by the impact that this pandemic has had on the routines of individuals. In general, social confinement and working and studying virtually have caused this population to remain more sedentary than it is normally (10). A meta-analysis conducted to evaluate the factors involved in the relationship between physical activity and depression showed a biological component (1). Also, physical activity decreases stress levels, and attending the gym or participating in exercise groups provides new social connections. Hence, the importance of promoting physical activity directly impacts physical and mental health and contributes to increasing the levels of social capital, which in turn is a protective factor against the development of clinically relevant depressive symptoms (35).

Analysis of the paired data showed that tobacco use was found to increase the risk of depression. A study of Korean college students showed that smoking was associated with depression and suicidal behavior, at least partly because adolescents are more vulnerable to nicotine sensitivity, which is associated with effects on cognitive function (40). In this study, a decrease in alcohol and tobacco consumption was suggested in students from wave 1 to wave 2; this could be explained because, during the confinement, establishments were closed, such as restaurants, discos, where young people were accustomed to drinking alcohol regularly (41). Also, as evidenced in this study, most students had to return to live at home with their parents (29.16%), which also explains the decrease in tobacco consumption. In this sense, the influence of context on substance use has been investigated, and contextual changes, such as “moving back home,” could represent an opportunity to facilitate cessation (42).

On the other hand, getting a good night's sleep was a protective factor for the development of depression. It has been shown that sleep, especially in young people, is fundamental for maintaining normal body functions. So young people with poor sleep will negatively impact their neurocognitive functions and emotional wellbeing, which can aggravate academic difficulties (43). Thus, it is important to promote the importance of a healthy lifestyle for its potential to prevent the development of depression (44).

The World Health Organization states that mental disorders are important causes of morbidity and mortality globally, noting that one in four people will develop a mental disorder at some point in their lives (1). It is also essential to highlight the impact that the COVID-19 pandemic has had on the general population. It altered the daily routines of all people globally, which is reflected in the observed increase in mental illnesses and unhealthy lifestyles during the pandemic (45).

We discovered other interesting findings when comparing the results of wave 1 with those of wave 2. Almost 30% of the students returned to live at home with their parents due to the pandemic. Of this group, nearly 38% reported depressive symptoms, which was lower than those who lived at a relative's home, college residence, or in a rented house. A survey conducted among university students in India reported that living at home with parents favored mental health due to the family environment and good relationships with parents (10). In contrast, students who move out of their family homes have to socialize with new people and establish a new social network, which may increase their stress levels and affect their mental health (9).

Our findings suggest that the mental health of university students may have been substantially affected during the COVID-19 pandemic.

## Implications

Our study shows significant findings on the mental health of Universidad de Los Andes students and their health behaviors. It also shows the value of mental health protective factors, such as social capital. It is necessary to provide opportunities that favor social interaction and promote the creation of interest groups among students, thus strengthening their support networks and levels of social capital. In turn, encourage the practice of sports or activities that promote a healthy lifestyle and help with stress management, positively impacting the mental health of these young people.

In addition, the COVID-19 pandemic has impacted the lives of young adults across the globe. Before the pandemic, university students comprised a population considered particularly vulnerable to mental health problems (36). The COVID-19 pandemic has brought unprecedented stress to students. Our findings corroborate recent studies that high levels of social capital have contributed to some populations being less affected by the measures used to cope with this pandemic (10, 11, 46).

Universidad de los Andes has some general guidelines to promote student wellness. It has a variety of services available, such as counseling, advising, academic support, and leisure time strategies focused on positively impacting the health and wellbeing of students. In this institution, a culture of wellness is promoted throughout the campus. The directors and professors are trained in mental health issues not to diagnose and prescribe but to operate under a wellness mentality in every interaction and detect risks that can be referred to on time. Increasing the availability of places that favor socialization and the practice of sports or other activities that promote a healthy lifestyle will remain fundamental to mitigate the impact of the transition to university life, in this case, aggravated by the current pandemic in our country and other university settings.

Furthermore, we need additional studies that evaluate the social capital in Latin American contexts to help determine the causes of poor health outcomes. We also need studies that contribute to developing and implementing interventions that strengthen social capital in different communities and strategies to promote healthy lifestyles in this population.

## Strengths and Limitations

This study has some limitations that must be acknowledged. First, the study and data collection fell right into the pandemic, and therefore, different recruitment approaches had to be applied. While the first wave was conducted in person during January and February 2020 and directly on the university campus, the second wave, which took place in August 2020, had to be entirely online. This has resulted in a much lower response rate than expected. Therefore, the results of longitudinal changes have to be interpreted with caution and take into account the potential loss of statistical power in light of the valuable information collected during an unprecedented global public health crisis such as the COVID-19 pandemic. Second, self-reported measures were used concerning questions investigating lifestyles and depressive symptoms, and we cannot exclude recall bias.

Despite these limitations, the present study has important strengths. It should be emphasized that our study is not a cross-sectional study, but uses a two-wave panel design that allows us to make early inferences about the directionality of the effects of social capital on mental health. Furthermore, this is the first study assessing the presence of clinically relevant depressive symptoms and the relationship with levels of social capital, perceived stress, and lifestyles among university students both before and during the COVID-19 pandemic. Therefore, the findings offer important information about the value of social capital and the importance of maintaining lifestyles during times of crisis.

## Conclusions

Our study showed relevant findings on the role of social capital in preventing depressive symptoms and promoting healthy lifestyles in this population. This study found that the proportion of first semester university students with clinically depressive symptoms was already elevated before the COVID-19 pandemic and may have increased even further during the pandemic, particularly among those with lower levels of social capital. Thus, students' health has to be prioritized and put at the top of public health agendas, and the implementation of measures strengthening social capital should be considered.

## Data Availability Statement

The data set of this study is available upon request to the corresponding author.

## Ethics Statement

The Ethics Committee of the Universidad de los Andes approved this study (act number: 201909242). Written informed assent or consent was obtained, depending on whether participants were adolescents or young adults. Participants were guaranteed confidential treatment of their data and were given the possibility to withdraw from the study at any time. When participants reported having relevant depressive symptoms or suicidal ideas, they received psychological support from the university health services.

## Author Contributions

LS, PS, and AR collected data at Universidad de los Andes (Colombia). LS, PS, AR, JG, and IB conducted the statistical analyses. All authors participated in revising the results and the manuscript and approved the final version.

## Conflict of Interest

The authors declare that the research was conducted in the absence of any commercial or financial relationships that could be construed as a potential conflict of interest.

## Publisher's Note

All claims expressed in this article are solely those of the authors and do not necessarily represent those of their affiliated organizations, or those of the publisher, the editors and the reviewers. Any product that may be evaluated in this article, or claim that may be made by its manufacturer, is not guaranteed or endorsed by the publisher.
